# Anthropogenic Disturbance and Biodiversity of Marine Benthic Communities in Antarctica: A Regional Comparison

**DOI:** 10.1371/journal.pone.0098802

**Published:** 2014-06-11

**Authors:** Jonathan S. Stark, Stacy L. Kim, John S. Oliver

**Affiliations:** 1 Terrestrial and Nearshore Ecosystems Program, Australian Antarctic Division, Kingston, Tasmania, Australia; 2 Moss Landing Marine Laboratories, Moss Landing, California, United States of America; Northwest Fisheries Science Center, NOAA Fisheries, United States of America

## Abstract

The impacts of two Antarctic stations in different regions, on marine sediment macrofaunal communities were compared: McMurdo, a very large station in the Ross Sea; and Casey, a more typical small station in East Antarctica. Community structure and diversity were compared along a gradient of anthropogenic disturbance from heavily contaminated to uncontaminated locations. We examined some of the inherent problems in comparing data from unrelated studies, such as different sampling methods, spatial and temporal scales of sampling and taxonomic uncertainty. These issues generated specific biases which were taken into account when interpreting patterns. Control sites in the two regions had very different communities but both were dominated by crustaceans. Community responses to anthropogenic disturbance (sediment contamination by metals, oils and sewage) were also different. At McMurdo the proportion of crustaceans decreased in disturbed areas and polychaetes became dominant, whereas at Casey, crustaceans increased in response to disturbance, largely through an increase in amphipods. Despite differing overall community responses there were some common elements. Ostracods, cumaceans and echinoderms were sensitive to disturbance in both regions. Capitellid, dorvelleid and orbiniid polychaetes were indicative of disturbed sites. Amphipods, isopods and tanaids had different responses at each station. Biodiversity and taxonomic distinctness were significantly lower at disturbed locations in both regions. The size of the impact, however, was not related to the level of contamination, with a larger reduction in biodiversity at Casey, the smaller, less polluted station. The impacts of small stations, with low to moderate levels of contamination, can thus be as great as those of large or heavily contaminated stations. Regional broad scale environmental influences may be important in determining the composition of communities and thus their response to disturbance, but there are some generalizations regarding responses which will aid future management of stations.

## Introduction

Understanding the ecological consequences of disturbance from Antarctic stations is important in the management and prevention of environmental impacts. A total of 31 countries operate 82 seasonal or year-round research stations in Antarctica [1], the majority of which are located in coastal areas [2], [3], however, currently there is little known of the impacts of these stations on marine environments. Clearer understanding of the way in which Antarctic coastal ecosystems respond to disturbance, together with proven methods for detecting impacts and assessing biodiversity, will aid operators and policy makers attempting to improve environmental management of research stations. Although the response of benthic communities to disturbance has been well studied in tropical and temperate regions, these cannot necessarily be generalized to polar regions. Countries managing stations need the ability to easily assess their potential impacts and understand them in a Antarctic-wide context. This includes assessing local biodiversity in comparison to regional and Antarctic wide biodiversity and how it is impacted. An understanding of the relative size or nature of an impact will aid in decisions regarding its management or remediation. The remediation of environmental damage, and marine spatial protection and management, have both been identified as high priority issues by the Committee for Environmental Protection (CEP) of the Antarctic Treaty Consultative Parties (ATCP) [4]. The need for assessment of biodiversity in regional planning has also been highlighted by recent international endorsements of marine protected areas (MPA) as a management tool, such as by the World Summit on Sustainable Development [5] and the World Parks Congress [6]. The Commission for the Conservation of Antarctic Marine Living Resources (CCAMLR), together with the CEP, have made a commitment to work towards creation of an MPA network in Antarctica. This study aims to increase our understanding of the way in which Antarctic coastal benthic communities respond to anthropogenic disturbance and improve approaches to the detection and assessment of the size and nature of impacts of Antarctic stations. We also examine the regional assessment and comparison of biodiversity, a key component in MPA planning.

Most stations are located in coastal ice free areas [2], [3], which are biologically important as habitats for terrestrial plants (e.g. moss and lichen), breeding birds (e.g. penguins and petrels) and highly diverse coastal marine ecosystems, particularly of benthic communities [7], [8]. Coastal ice-free land comprises less than 0.01% of the continent [9] and correspondingly, coastal shallow water marine ecosystems are rare in Antarctica. Shallow water habitats are isolated by large expanses of continental ice, glaciers, ice shelves and deep water and can be considered as “islands” in their lack of connection to each other. Genetic research indicates highly limited connectivity between these areas for some species [10]–[12]. Activities associated with stations known to cause environmental impacts include fuel and oil spills, landfill and marine waste disposal sites, and sewage and wastewater disposal. As stations are generally situated very close to the coast for ease of access, these activities can cause impacts on marine benthic communities [13], [14]. Thus environmental impacts in these areas, even if only local in scale and limited to the vicinity of Antarctic stations, will have implications greater than might at first appear to be the case in an Antarctic-wide context. This is due to stations being located in habitat that is rare, isolated and biologically important. Antarctic benthic communities are particularly vulnerable to environmental impacts due to their slow recovery from disturbance, the high proportion of brooding and poorly dispersing species [12], and the highly variable spatio-temporal nature of primary production [15]. Many stations have been established for up to 50 years and with the number of bases and tourism on the increase these ecosystems will come under increasing pressure. Antarctic marine ecosystems are forecast to be particularly vulnerable to climate change [14], [16]–[18], which may further exacerbate potential impacts of stations.

Environmental impact research in Antarctic coastal marine habitats has been done extensively at two stations: McMurdo (USA) in the Ross Sea; and Casey (Australia) in East Antarctica. These stations are of similar ages, established around the International Geophysical Year (IGY) in the late 1950’s, however, in most other aspects they are very different. McMurdo is atypical of Antarctic stations: it is very large (population 200–1500 people), it has had activities not found elsewhere in Antarctica (e.g. a nuclear power station, military base) and in places it is as highly polluted as industrial harbours in other parts of the world [19]–[22], and in these aspects it is unique. Casey is more representative of the majority of stations: it is small (15–75 people) with less infrastructure, and pollution levels are much lower than McMurdo [23], [24]. Marine environmental impacts have been detected at both stations [13], [23], [25]–[29]. Environmental impact and biodiversity assessments are expensive and time consuming, particularly in Antarctica. Antarctic marine benthic research is generally fragmented, patchy in distribution, limited in extent and focused on small areas adjacent to or nearby each nation’s area of operations or station locations. This is due to the isolation of Antarctic stations on “islands” of ice-free rock and their operation by different countries. Opportunities to sample widely around Antarctica concurrently, using the same methods and sampling design, are unlikely and thus the only realistic way comparisons of regional response to human impacts can be made is with existing data from different studies [30]. Comparing data from different studies is hampered by a range of issues as research is conducted for different reasons by scientists from different countries, using differing methodologies, sampling designs, processing and identification, and at different spatial and temporal scales [30]–[32].

Measurement of diversity, commonly recorded as species richness, is central to ecological theory and conservation strategies and is affected by spatial scale and sampling effort [31], [33], [34]. Description and comparison of community diversity is best done in a well defined spatial framework [35], [36]. Some of the difficulties in comparing biodiversity assessments from different studies can be avoided or reconciled by using species-area relationships and species accumulation and rarefaction curves [30], [31]. Comparing species-area curves from different studies requires a common or standardized sampling unit [34], [37]. Sample based and individual based rarefaction methods allow the meaningful comparison of standardized datasets [31], [33]. Thus when attempting comparisons it is vital to understand the limitations inherent in the data and its interpretation. Some of these issues associated with comparing biodiversity estimates from different studies have been examined by various authors [32]–[34], [37], [38].

This study compares soft-sediment infaunal communities near McMurdo and Casey using previously unrelated studies to explore the nature and extent of responses of soft-sediment communities to anthropogenic contamination and disturbance in Antarctica. We examine issues of methodological bias, sampling effort, taxonomic uncertainty in comparisons of different studies, and effects of spatial scale on measures of biodiversity. We describe responses to anthropogenic disturbance of communities at species and higher taxonomic levels, effects on biodiversity, identify potential indicator organisms and compare the relative size of impacts at each station. This information will enable more rapid assessment and early detection of environmental impacts at other Antarctic Stations, leading to improved environmental management.

## Materials and Methods

**Figure 1:**
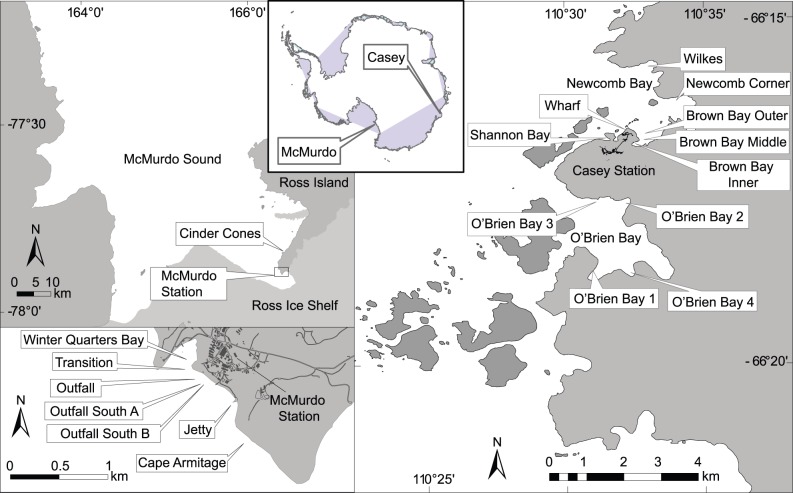
Location of sampling sites around McMurdo and Casey Stations.

Casey station is situated in the Windmill Islands in East Antarctica at 66° 17′S, 110° 32′E (Fig. 1). The data used for the Casey region comes from a survey done in the summer of 1997/98. Sampling design and details can be found in Stark et al. [28]. In summary: samples were taken by divers using hand held cores (10 cm diam.×15 cm long), inserted into the sediment up to 10 cm; samples were taken from 9 locations over an 8 week period between October and December 1998 with a hierarchical spatially nested sampling design with 3 scales (plots = 10 s of metres, sites = 100 s of metres and locations = 1000 s of metres); 16 samples were taken at each location (4 replicates within a 2 m diam. plot, 2 plots within a site, 2 sites within a location); one location was sampled twice over the sampling period; there were a total of 160 samples used in this data set (Table 1); samples were sieved on a 1 mm mesh and sorted; a subset (4 locations) were sieved and sorted at 0.5 mm; taxa were identified to species, or morphospecies where identifications were uncertain or could not be reliably made.

**Table 1:** Details of samples used in this study from each region.

McMurdo locations	Classification	Years sampled								Depth (m)	n
Cape Armitage	Control	1988	1990		1992	1993			1998	6, 9, 12, 18	54
Cinder Cones North	Control	1988						1997	1998	18, 21	20
Jetty	Control	1988	1990		1992	1993	1996	1997	1998	18	42
Outfall South A	Intermediate		1990	1991	1992	1993			1998	9, 18	36
Outfall South B	Intermediate	1988	1990		1992	1993			1998	18	29
Outfall	Disturbed	1988	1990	1991	1992	1993			1998	9, 18	42
Transition	Disturbed		1990		1992	1993			1998	18	24
Winter Quarters Bay	Disturbed	1988	1990	1991	1992	1993			1998	18, 24	78
**Casey locations**											
Brown Bay Inner	Disturbed								1998	6 to 10	16
Brown Bay Middle	Disturbed								1998	10 to 15	32
Wharf	Disturbed								1998	6 to 10	16
Brown Bay Outer	Intermediate								1998	15 to 20	16
Shannon Bay	Intermediate								1998	15 to 20	16
Wilkes	Intermediate								1998	10 to 15	16
O’Brien Bay-1	Control								1998	10 to 15	16
O’Brien Bay-2	Control								1998	15 to 20	16
O’Brien Bay-3	Control								1998	10 to 20	16

Summary of samples taken at each station that were used in comparisons in this study. Classification refers to nominal level of disturbance at each station, based on metals, TOC, TPH and organic contaminants in sediments and site history; n = total number of samples at each location.

McMurdo Station is situated on Ross Island at 77° 51′S, 166° 40′E in the Ross Sea. The data used for McMurdo comes from a ten year period of sampling from 1988 to 1998. Sampling details can be found in Lenihan and Oliver [13]. In summary samples were taken by divers with a hand held corer (10 cm diameter) from 8 locations, generally with a minimum of 6 replicate cores per location with some locations sampled at several water depths (Table 1). In total there were 325 samples used in this data set. Samples were sieved on a 0.5 mm mesh and identified to species or morphospecies where identifications were uncertain or could not be reliably made.

All necessary permits were obtained for the described field studies. Collections from Casey were approved by the Commonwealth of Australia under the Antarctic Marine Living Resources Act 1981, under permit AMLR 98/10. No special permits are required for invertebrate collections from McMurdo.

### Biodiversity

In this study we compare numerical species richness within a specified spatial framework, which takes into account sampling effort and the spatial extent of sampling, which have been shown to be important in comparisons of biodiversity [30], [34]. We have used the following terminology from Gray [36] to distinguish spatial scales of comparison and sample size, relating to numerical species richness (SR).

- Point species richness: SR_P_ The species richness (number of species) of a single sampling unit.
- Sample species richness: SR_S_ The species richness of a number of sampling units from a site of defined area.
- Large area species richness: SR_L_ The species richness of a large area which includes a variety of habitats and assemblages.
- Habitat species richness: SR_H_ The species richness of a defined habitat.

We also used species-area curves to compare species richness in both regions and at control and disturbed locations. In addition to species accumulation curves based on the number of species observed we employ several non-parametric asymptotic estimators of species richness (Chao-2, Jacknife-1 and Jacknife-2) to estimate the likely lower-bounds of overall species richness in each region. These methods are currently regarded as the most suitable for estimating the minimum number of species in an assemblage [33], [39] and are particularly useful where the observed species richness curves have not reached an asymptote [31]. Species accumulation/rarefaction curves were calculated using the program PRIMER (V 6.1.13).

A wide variety of indices have been used to describe the two components of species diversity: richness and evenness. We use observed species richness and species-area plots, which show the actual number of species living in a range of comparable areas, regardless of any statistical relationships between the number of species and number of individuals. We have chosen not to use the commonly utilised index H’ (the Shannon-Wiener index) due to well documented criticism of the difficulty in biological interpretation [35], [40]–[42] and non-suitability for environmental quality assessment [43]. We have also avoided use of the evenness index J as it is derived from H’. Instead we employ Simpson’s index, λ, [44] as it is relatively unaffected by sample size, is more intuitively meaningful and has greater utility in a range of contexts, as reviewed by Magurran [42] and assessed by other authors [35], [45]–[47].

### Sampling Bias from Mesh Size

**Figure 2:**
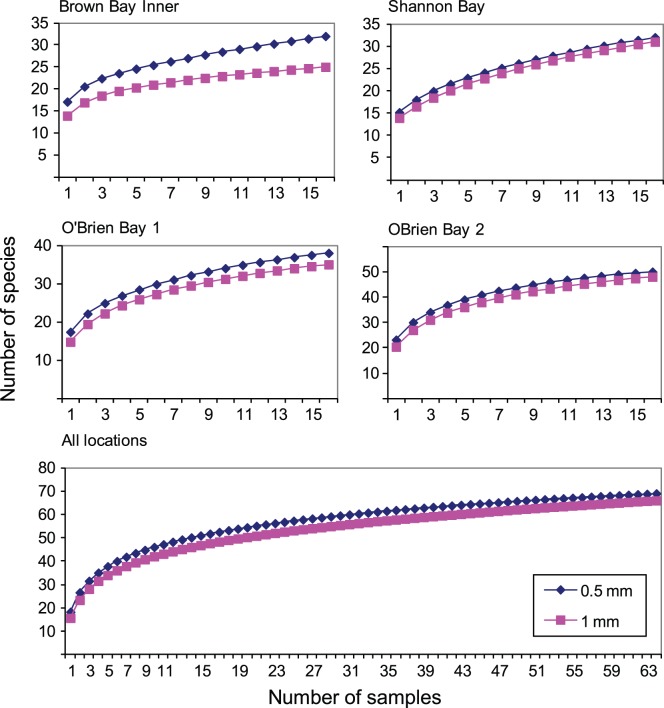
Effect of mesh size on number of species. Species accumulation (species-area) curves comparing 0.5 mm with 1 mm mesh size for replicate cores at 4 locations at Casey Station and for all four combined.

The potential effects of using a 1 mm sieve as opposed to a 0.5 mm sieve are underestimates of species richness and abundance in samples sieved using a 1 mm screen. We compared 4 locations (16 replicates per location) at Casey using both mesh sizes. Abundance was on average 1.49 (0.11 SE) times greater per sample in 0.5 mm samples (range: 1.21–1.75). Species richness was on average 1.17 (0.03 SE) times greater on a per sample basis using a 0.5 mm mesh (range: 1.11–1.25). When species richness was considered cumulatively, per location (4 locations, n = 16 per location), this effect was reduced with a mean of 1.10 (0.06 SE) times greater number of species per location using a 0.5 mm mesh (Fig. 2). This effect of mesh size was further reduced when species richness was calculated cumulatively over all 4 locations (n = 64), with only 1.05 times more species on a 0.5 mm mesh (Fig. 2). Thus, as the sample size increases, the difference in the cumulative number of species captured on a 1 mm vs. 0.5 mm mesh decreases. On an even larger data set, such as the 160 samples from Casey used in this study, we assume that the sample bias on species richness would be negligible.

### Taxonomic Uncertainty

In many regions of the world, particularly remote and difficult to access areas such as Antarctica, there remains great taxonomic uncertainty and a paucity of taxonomic resources such as identification keys. In addition, modern taxonomic tools such as DNA sequencing are revealing a wide degree of cryptic speciation among otherwise nearly identical species, and there are numerous examples from Antarctica [10], [48], [49]. A large degree of uncertainty exists in identification of whole communities of taxa to species level when comparing species sampled in different studies, without the direct matching of actual specimens from different regions, particularly when morphospecies identifications are used when species cannot be reliably identified. We did not compare community patterns in the same analysis using species level identifications. The infaunal species from each station were identified by different scientists in different countries and no direct comparisons of individual species were made to confirm the same identities. To overcome this, a higher level of taxonomic resolution was used, as there is far less uncertainty in identifications at higher levels. In comparisons of assemblage patterns and their response to anthropogenic impacts, the two assemblages were directly compared at the level of family, and for some taxa a higher taxonomic unit was used such as order or class. Thus the assemblage response to disturbance in each region is presented at the family level. This has been shown to be as effective as species level identifications for detecting impacts [50], [51].

### Disturbance

We define disturbance for the purposes of this paper as being of anthropogenic origin, such as pollution. We did not include natural disturbances such as ice scour, as the sites used in this study were selected, within each region, to be as similar as possible with respect to physical characteristics such as duration of ice cover and exposure to ice scour. The sites used in this study are ice covered for most of the year, being ice free for only short periods of 2 to 8 weeks in late summer (e.g. [52]) and with some sites ice covered year round in some years. Thus they are largely protected from the impacts of ice scour from floating sea ice and ice bergs. In addition, no sites were shallower than 6 m depth or within 50 m of shore and thus not in the zone of ice foot scour e.g. [53].

Casey and McMurdo have very different histories, although both areas have been occupied continuously for a similar period. Different levels of contamination have occurred at each station, largely a result of their different sizes, but the general types of activities at each station are similar such as waste disposal, hydrocarbon contamination from leaks and spills, and sewage disposal. Waste disposal sites located on or very close to the shoreline were perhaps the most significant source of contamination at both stations, but sewage outfalls are also a significant source of organic enrichment and contaminants at each station. Contamination of the marine environment has been well documented at McMurdo [13], [19]–[21], [25], [54], [55] and Casey [9], [23], [24], [28], [29], [56].

The disturbed sites at Casey include Brown Bay, which is adjacent to a waste landfill site that operated for a period of 22 years (1965–1986). Waste disposed here consisted of building materials, metal, waste oil, chemicals, machinery and equipment, electronic components and food wastes [57]. Waste was also bulldozed into the bay and this remains on the seabed in Brown Bay. The marine environment adjacent to the Casey Wharf has been subjected to a range of disturbance including a large fuel spill in 1991 [28], physical disturbance from the propeller wash of boats and the seabed is also strewn with rubbish and metallic items dropped from resupply vessels. A sewage outfall discharges secondarily treated wastewater into Shannon Bay and for some periods over summer the treatment system is bypassed due to limited treatment capacity. The site of Wilkes is adjacent to another abandoned waste disposal site at the former station of Wilkes, which operated from 1957 until 1969.

At McMurdo the disturbed sites include Winter Quarters Bay (WQB), the ice-dock site of fuel and cargo resupply activities, adjacent to the mechanical workshops as well as a landfill waste disposal site situated on the foreshore the bay which operated between 1955 and the late 1980’s. Waste was also doused with fuel and burnt on the site as well as being deposited onto the sea ice [58]. WQB has a long history of contamination and the McMurdo wastewater outfall is also a known source of organic enrichment and chemical contamination [19], [20], [26], [59]. The wastewater outfall discharged untreated effluent until 2003 when a secondary treatment plant was installed [60].

Data on contaminant levels in marine sediments are largely taken from other studies. Different methods were used to measure metals (Casey [61], McMurdo [19]) but comparison among stations takes methodological biases into account. At McMurdo a strong acid (conc. HNO_3_) digest was used on the sediments [19], while at Casey both a strong acid (conc. HNO_3_+ conc. HF) [61] and a weak acid (1 M HCl) partial digest were utilised [23].

Different methods were also used to measure organic carbon in sediments in each region. At Casey a loss-on-ignition method was used and a correction factor (division by 1.8) was applied to convert from total organic matter to give an estimate of % organic carbon [62]–[64], as organic carbon is closely correlated with weight loss on ignition. There is some controversy in the literature, however, regarding the appropriate conversion factor [65]–[67], and thus the value of 1.8 used here is intended to provide an approximation of the relative organic carbon concentrations and not an exact measure. Organic carbon in McMurdo sediments was determined by an elemental carbon analyser as detailed in [19]. The redox potential discontinuity layer (RPD) was estimated by divers while sampling at Casey sites. Citations for methods for all other contaminants measured are given where data are used.

A set of simple criteria were used to classify species as potentially useful indicators of anthropogenic disturbance in Antarctica: 1) Opportunist taxa – those consistently more abundant at disturbed sites than controls, or occasionally very dominant at disturbed and/or only low abundances at control; 2) Sensitive taxa - found commonly at control sites (but not necessarily abundantly) but not found at disturbed sites, or extremely rarely.

### Statistical Analysis

Simpson dominance, taxonomic diversity (TDΔ), taxonomic distinctness (TDΔ*) and average taxonomic distinctness (TDΔ^+^) were calculated according to [68], [69] using the program PRIMER V6.1.13. Differences in taxonomic diversity between control, intermediate and disturbed locations were tested using univariate PERMANOVA [70], [71], based on a Euclidean distance similarity matrix and a 2 factor design with disturbance (3 levels) and locations nested within disturbance for each region separately. Univariate PERMANOVA is a permutation based analogue of traditional ANOVA and gives equivalent results, but caters for unbalanced designs by offering a choice for the type of sums of squares used for partitioning, and there are no assumptions regarding the distribution of variables [72].

To test hypotheses regarding differences between macrofaunal communities at control, disturbed and intermediate locations, a range of multivariate methods were used including ANOSIM tests for differences between groups, distances between group centroids and MDS and PCO ordinations, which were all based on Bray-Curtis similarity matrices of square root transformed abundance data, calculated using PRIMER.

## Results

### Comparison of Anthropogenic Disturbance

**Figure 3:**
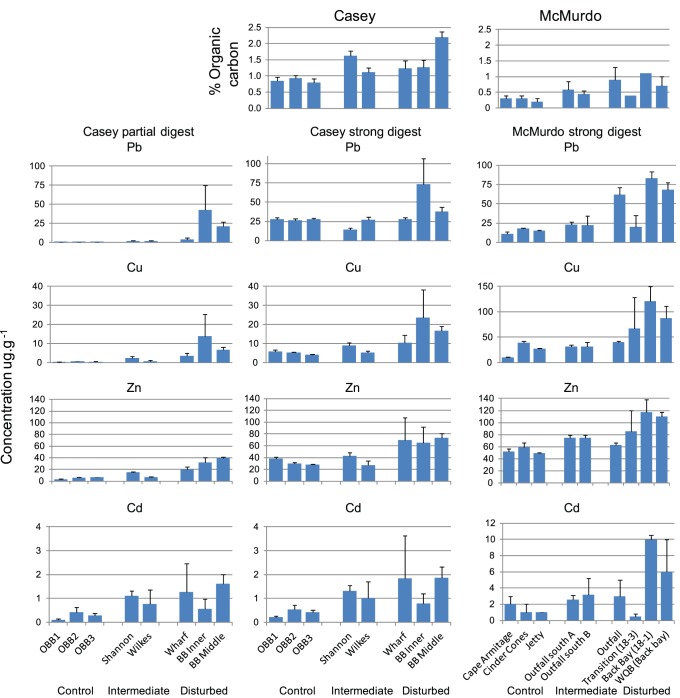
Contamination levels in sediments. Organic carbon content and metal concentrations in marine sediments at Casey and McMurdo Stations. Note different scales for copper (Cu) and cadmium (Cd) in each region. Data for Casey are from [61] and [23]; data from McMurdo are from [13], [19], [25]. Pb = lead; Zn = zinc; OBB = O’Brien Bay; BB = Brown Bay; WQB = Winter Quarters Bay.

Direct comparison of contaminant levels between the stations is difficult as different sampling, analytical and assessment methods have been used at each station, with only patchy measurement of some contaminants, particularly at Casey. At each station there is a range of contamination and individual sites can be nominally classified into groups which are broadly representative of their contamination status at each station (Table 1): control (uncontaminated background levels), intermediate (moderate local contamination in comparison to background or the highest local levels) or disturbed (highest levels of local contamination). For example, sediment metal levels show a pattern of the three groups (Fig. 3). We used these three broad contamination classifications (control, intermediate and disturbed) to compare the response of macrobenthic communities to anthropogenic disturbance at each station. We also combined the intermediate and disturbed groups together for some analysis in comparison with the control groups. These groups are only meant to be indicative of contamination levels against background (control) levels within each region and do not indicate similar levels of disturbance in inter-station comparisons. Contamination levels at Casey are generally lower and are not in the same range as McMurdo. The known history of each site was also used to contribute to the classification of sites into these 3 groups.

At Casey the most contaminated sites are those adjacent to the old waste disposal site in Brown Bay (Brown Bay Inner, Middle) and the Casey Wharf. The sites classified as intermediate disturbance at Casey are: Shannon Bay, adjacent to the wastewater outfall; Wilkes, adjacent to another abandoned waste disposal site; and Brown Bay Outer. Several control sites are used in comparison to these including four sites in O’Brien Bay and a single site in Newcomb Bay: Newcomb Corner (Figure 1, Table 1, Table 2).

**Table 2:** Other marine sediment contaminants measured at control and disturbed locations at Casey and McMurdo, expressed on a dry weight basis.

		TPH mg/kg	PAHs ug/kg	PCBs 1988 ug/kg	PCBs 2003 ug/kg	PBDEs uk/kg
**Casey**						
O’Brien Bay-2	Control	0 (0)^A^	NA	NA	NA	NA
O’Brien Bay-4	Control	73 (55)^A^	NA	NA	NA	NA
Newcomb Corner	Control	40 (17)^A^	NA	NA	NA	NA
Brown Bay Outer	Intermediate	145 (105)^A^	NA	NA	NA	NA
Brown Bay Inner	Disturbed	698 (241)^A^	NA	NA	NA	NA
Brown Bay Middle	Disturbed	318 (167)^A^	NA	NA	NA	NA
**McMurdo**						
Cape Armitage	Control	0^B^	1.75 (2.47)^C^	3.75 (2.33)^D^	0^C^	NA
Cinder Cones	Control	0^B^	0 (0)^C^	0.43 (0.52)^D^	0^C^	162^E^
Jetty	Control	0.5^B^	8.15 (11.5)^C^	43 (17)^D^	30.6^C^	43.6^E^
Outfall South A	Intermediate	NA	90 (0.57)^C^	NA	21.4^C^	760^E^
Outfall South B	Intermediate	NA	38 (2)^C^	NA	110.5^C^	NA
Outfall	Disturbed	1^B^	7618 (8298)^C^	345 (92)^D^	331.4^C^	3910^E^
WQB (18-3)/Middle	Disturbed	15^B^	39 (56)^C^	175 (35)^D^	146.8^C^	499^E^
WQB (18-1)/Inner	Disturbed	4500^B^	74 (67)^C^	1095 (431)^D^	20.6^C^	NA
WQB (Back bay)	Disturbed	2600^B^	NA	1400^D^	NA	1420^E^

Data taken from A [24], B [19], C this study, D [21], E [20]. TPH = total petroleum hydrocarbons; PAH = polycyclic aromatic hydrocarbons; PCB = polychlorinated biphenyls; PBDE = polybrominated diphenyl ethers; NA = data not available; numbers in brackets indicate standard deviation.

At McMurdo the most contaminated sites are those in Winter Quarters Bay (WQB) and adjacent to the sewage outfall. Two sites downstream of the outfall, Outfall South A and B are classified here as intermediate disturbance. Several control sites were used in comparisons including Cape Armitage, Cinder Cones and the station wharf (Jetty).

One of the most routinely assessed measures of environmental contamination is trace metals in marine sediments. Metals have been measured at both stations but with different methods, making direct comparison difficult. Strong acid digests break down mineral phases in the sediment and are often used because they are highly reproducible, however they typically only identify the most intense local contamination [61]. Some metals are frequently not identified as being above background when they may in reality be anthropogenically elevated, producing a Type II statistical error. A weak acid partial extraction gives an estimate of the bioavailable metal fraction in marine sediments and targets the labile mineral phases, sorbed to grains or in the pore water [61], [73], [74]. A strong acid extraction will give much higher results than a weak acid partial-extraction, up to and greater than an order of magnitude difference depending on the metal measured [61].

A comparison of lead, copper, zinc and cadmium concentrations from Casey and McMurdo demonstrates the nominal classification into control, intermediate and disturbed groups at each station (Fig. 3), and also shows the difference when using the strong or partial digests at Casey. Thus the strong acid digest may obscure low levels of anthropogenic contamination at McMurdo. Direct comparison of strong digests between regions shows that McMurdo (HNO_3_) has greater concentrations of metals at contaminated sites than at Casey contaminated sites, even though a stronger digest was used at Casey (HNO_3_+ HF). While metal concentrations appear higher, a proportion of this would be metals tightly bound in the mineral matrix and not available to biota. However, even taking this into consideration concentrations are higher at McMurdo, for example, lead at contaminated sites is up to ∼5x the controls, while at Casey using a strong digest it is only ∼3x the controls (Fig. 3). This is likely to be an underestimate of the difference between the two stations as a stronger digest was used for Casey than McMurdo. Using partial digest data from Casey, lead at disturbed sites is up to ∼180x background levels.

The contamination at both stations also includes hydrocarbons and organic contaminants [21] (Table 2). Although the same contaminants were not measured at both stations, these data provide further evidence of the nominal disturbance classifications within each region. For example the Casey outfall at Shannon Bay has higher levels of organic carbon, copper and cadmium than controls (Fig. 3, Table 2). There is no organic contaminant data available from Casey, although some preliminary sampling indicates polybrominated diphenyl ether (PBDE) levels at the outfall site, Shannon Bay, are well above background (unpublished data). PBDEs were detected in wastewater effluent and marine sediments around Davis station, another Australian Station of similar size and history to Casey [75]. Disturbed sites at McMurdo have a range of other contaminants including PCBs (WQB and outfall) and PBDEs at the outfall (Table 2).

The other major form of contamination at each station is organic enrichment, primarily from sewage outfalls. The quantity of organic carbon in sediments is greater overall at Casey than at McMurdo when control sites are compared (Fig. 3). While the methods used in each region to determine carbon content are different, they are unlikely to be the cause of the difference between regions. At control sites at Casey carbon levels are 3 to 4 times higher than at McMurdo. Enrichment can be seen at the disturbed sites in both regions and is highest at the outfall at McMurdo and also high at the site of Casey’s outfall (Shannon Bay) and in Brown Bay adjacent to the former waste disposal site (Fig. 3). The redox potential discontinuity layer (RPD) at Brown Bay and Shannon Bay were also very shallow (<1 cm), while at the control sites they were deeper (≥5 cm), indicating anaerobic conditions and further evidence of elevated organic carbon content. The organic enrichment factor caused by the sewage outfall at each station is similar, ca. 3× background at McMurdo and ca. 2× background at Casey, with ca. 3× at the waste disposal site (Fig. 3).

### Comparison of Biodiversity

**Figure 4:**
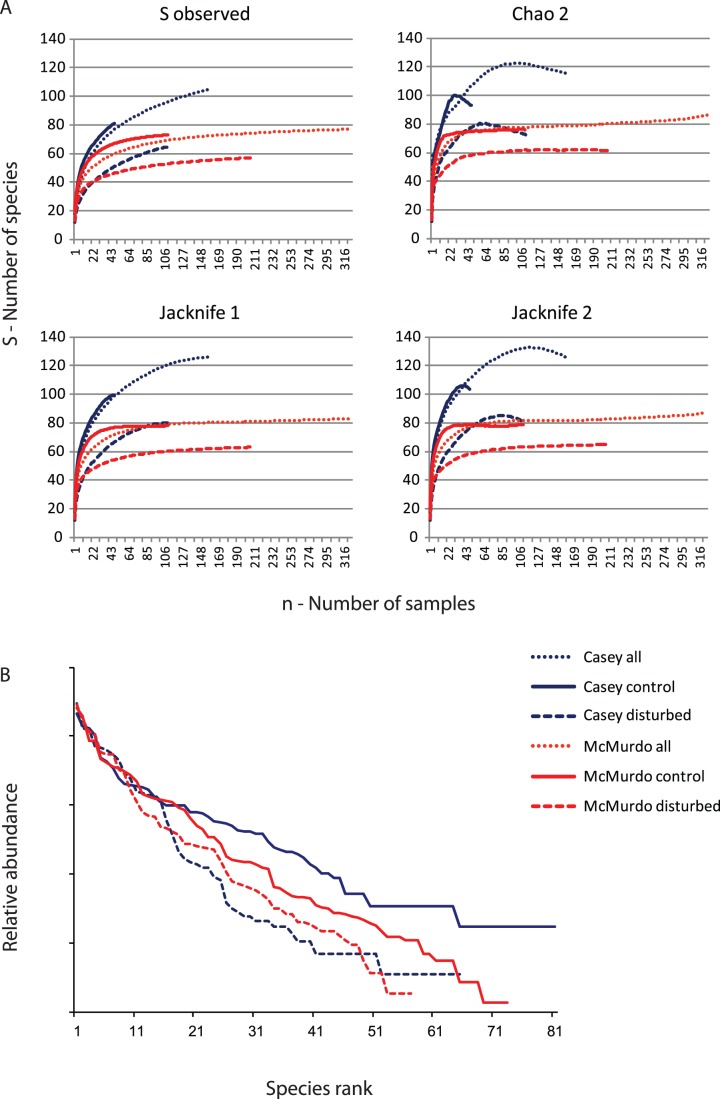
Patterns of biodiversity at Casey and McMurdo. A) Species accumulation curves for Casey and McMurdo regions, and control and disturbed locations within each region. B) Rank/abundance plots for macrofaunal communities from control and disturbed locations at Casey and McMurdo.

Observed species richness (S-obs) is greater at Casey than McMurdo, despite lesser sampling effort, but neither set of curves has reached an asymptote. (Fig. 4A). There is a very clear difference in S-obs between control and disturbed locations at both stations, and the disturbed locations at McMurdo have the lowest species richness overall. Sampling effort is also greater at disturbed locations in both regions. Non-parametric estimators of species richness were also used to compare each region. All three methods, Chao-2, Jacknife-1 and Jacknife-2 show similar patterns to S-obs, but with a greater total number of species, with estimates of 120 to 130 at Casey and 80 to 90 species at McMurdo (Fig. 4A). These non-parametric estimators are influenced by the sampling design, as seen in the declining smoothed curves for Chao-2 and Jacknife-2, a result of the fact that the implicit assumption of these methods, that all samples are effectively interchangeable within the region, is not actually true. Because these are heterogeneous sets of locations (within each region), once a certain number of samples have been reached (around 100), further samples are not from new locations but are replicates from within locations and the encounter rate of new species declines sharply. The spatially hierarchical nature of the sampling design, particularly at Casey (Locations, Sites, Plots, replicates within Plots), illustrates the difference between locations in the number and identity of species.

**Figure 5:**
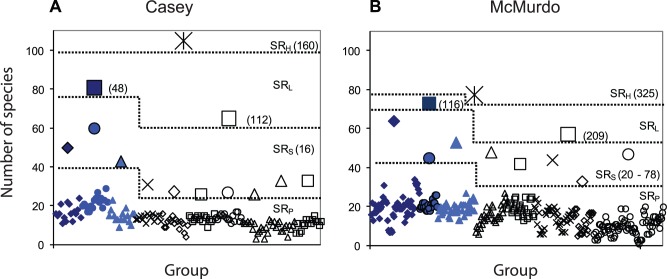
Species richness and spatial scales. Species richness at increasing spatial scales in each region. Different symbols represent different locations, blue symbols represent control locations, clear symbols disturbed locations, star symbol represents largest scale in each region (SR_H_, control and disturbed combined). Numbers in brackets indicate number of replicates in sample. SR_P_ = Point (replicate) species richness, SR_S_ = sample (combined replicates for location) species richness, SR_L_ = large sample species richness, SR_H_ = habitat species richness.

This effect of spatial scale can also be seen in a comparison of the observed number of species found at increasing spatial scales, from replicates to region. At the smallest scale, the point scale (replicate cores within a location) species richness (SR_P_) was variable and lower at Casey than McMurdo (Fig. 5, Table 3), but at this scale the comparison is biased by the underestimate at Casey due to the 1 mm sieve used. This difference is reduced when SR is examined at the sample scale (SR_S_ - replicates pooled within each location) and nullified at the large sample scale (SR_L_) (Fig. 5). At the habitat scale (SR_H_) Casey surpasses McMurdo (Fig. 5). This is an understimate biased by the lesser total sampling effort at Casey. Species richness was also greater at control than disturbed locations in both regions, which was more apparent above the point scale.

**Table 3:** Diversity, abundance and dominance of infaunal communities in each region.

Casey		Mean per Point (replicate core) ± SE			Sample pooled	
Control	*n*	SR_P_	Abundance	λ_P_	SR_S_	λ_S_
O’Brien Bay-1	16	16.6±0.9	76.7±7.2	0.15±0.02	50	0.11
O’Brien Bay-2	16	21.7±0.8	198.9±21.4	0.18±0.01	60	0.15
O’Brien Bay-3	16	14.8±0.9	85.6±7.0	0.24±0.01	43	0.20
**Disturbed**						
Shannon	16	13.9±0.4	301.6±8.3	0.33±0.02	31	0.31
Wharf	16	10.8±0.8	217.6±21.9	0.38±0.04	27	0.26
Wilkes	16	13.4±0.4	428.6±38.9	0.19±0.01	26	0.16
Brown Bay Inner	16	14.2±0.4	317.2±31.5	0.19±0.01	27	0.15
Brown Bay Middle	32	9.2±0.5	149.6±15.6	0.29±0.02	40	0.20
Brown Bay Outer	16	10.8±0.6	229.3±31.0	0.29±0.01	33	0.27
**Control mean (3 locations)**	48	17.7±0.7	120.4±13.7	0.19±0.02	51.0±4.9	0.15
**Disturbed mean (6 locations)**	112	11.7±0.3	274±26.6	0.27±0.02	30.7±2.2	0.22
					SR_L_	λ_L_
**Control pooled**					81	0.10
**Disturbed pooled**					65	0.14
**McMurdo**						
**Control**						
Cape Armitage	48	21.0±0.7	849.3±86.9	0.28±0.01	64	0.17
Cinder Cones	20	20.9±0.5	946.6±73.0	0.17±0.01	45	0.15
Jetty	42	18.9±0.5	323.7±20.3	0.15±0.01	53	0.11
**Disturbed**						
Outfall South A	36	15.7±0.8	384.7±61.7	0.25±0.02	48	0.19
Outfall South B	29	19.2±0.7	267.7±18.6	0.19±0.01	42	0.13
Outfall	42	14.3±0.8	467.2±73.6	0.25±0.02	44	0.22
Transition	24	12.3±1.0	244±16.6	0.36±0.04	33	0.16
Winter Quarters Bay	78	9.8±0.5	91.7±8.5	0.35±0.02	47	0.13
**Control mean (3 locations)**	116	19.6±0.4	706.5±66.6	0.20±0.01	54.0±5.5	0.14
**Disturbed mean (5 locations)**	209	13.3±0.4	291.1±44.5	0.28±0.03	42.8±2.7	0.17
					SR_L_	λ_L_
**Control pooled**					73	0.13
**Disturbed pooled**					57	0.15

Diversity, abundance and dominance for Casey and McMurdo infaunal communities. Note that a different sieve mesh size was used at Casey (1 mm) than McMurdo (0.5 mm). n = number of samples in group, SR_P_ = numerical species richness per core, SR_S_ = numerical species richness per sample group, λ = Simpsons diversity index.

**Figure 6:**
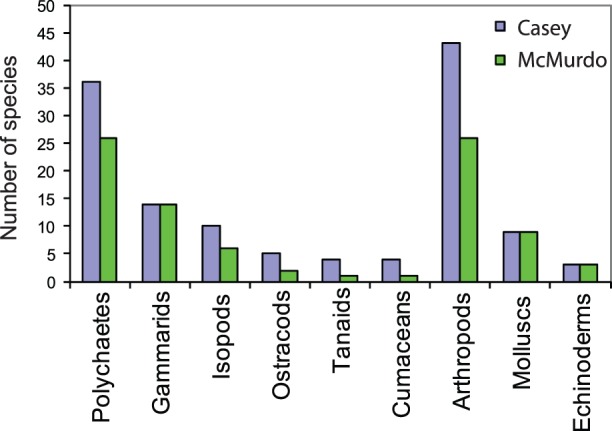
Diversity of major taxonomic groups. Comparison of taxonomic diversity in each region, based on the total number of species found in cores, McMurdo n = 325, Casey n = 160.

A comparison of species richness by major taxonomic groups indicates some clear differences between Casey and McMurdo. At Casey there are more species of polychaetes and most arthropod groups including isopods, ostracods, tanaids and cumaceans than at McMurdo, but no difference in the number of gammarid, mollusc or echinoderm species (Fig. 6).

**Figure 7:**
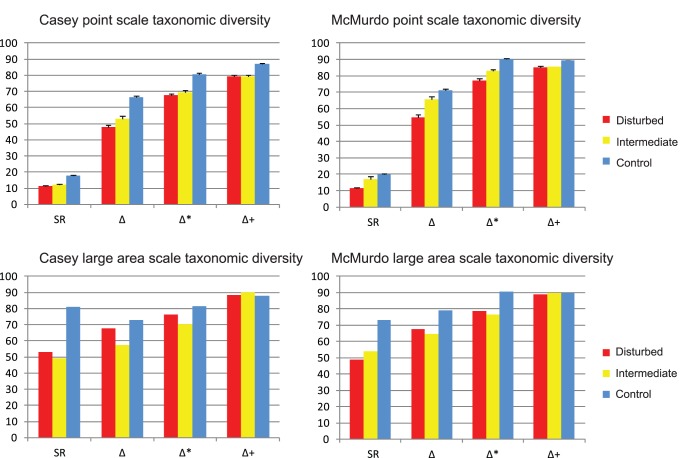
Taxonomic distinctness at different spatial scales. Comparison of taxonomic distinctness indices in each region. SR = species richness, Δ = taxonomic diversity (takes account of species abundances), Δ* = taxonomic distinctness, Δ+ = average taxonomic distinctness (based on presence-absence only), which equates to the average taxonomic distinctness between two randomly selected species.

Taxonomic diversity (Δ), taxonomic distinctness (Δ*) and average taxonomic distinctness (Δ^+^) were in most cases significantly greater at control than disturbed locations (ANOVA: p = 0.0001) in both regions, at both the average point scale and pooled over the large area scale (Fig. 7, Table 4). In general controls were not significantly different from intermediate sites, nor were intermediate different from disturbed, with a few exceptions (Fig. 7). This was due to very large differences between locations within the control, intermediate and disturbed groups. However at the extreme ends of the disturbance gradient, controls had greater taxonomic diversity than disturbed locations. Also the Δ^+^ did not show an effect of disturbance at the large area scale (Fig. 7). This indicates that over all disturbed and intermediate sites the average taxonomic distance between two randomly selected species is not different. Areas classified as intermediate or disturbed were not significantly different from each other at either scale. The effect of sampling effort on species richness (S-obs) and taxonomic diversity (Δ) can be seen in the large difference in S-obs from the point to the large area scales, but with very little change in taxonomic distinctness indices from point to large area scales. Species richness (SR) was greater over the largest scale at Casey, however, taxonomic diversity indices were greater at McMurdo than Casey, indicating greater relatedness among species at Casey or a broader range of taxa at McMurdo.

**Table 4:** Effect of disturbance and spatial scale on taxonomic diversity.

	Casey					McMurdo				
Taxonomic diversity (Δ)	df	MS	Pseudo-F	P(perm)	P(MC)	df	MS	Pseudo-F	P(perm)	P(MC)
Disturbance	2	4490.60	5.08	**0.05**	**0.05**	2	8485.90	8.61	**0.03**	**0.02**
Location(Dist)	6	934.88	16.74	**0.0001**	**0.0001**	5	1064.30	4.24	**0.002**	**0.001**
Residual	151	55.83				311	250.88			
Total	159					318				
Pairwise tests	t	P(perm)	P(MC)			t	P(perm)	P(MC)		
Control, Disturbed	**3.50**	**0.04**	**0.02**			4.04	**0.05**	**0.01**		
Control, Intermediate	1.75	0.10	0.18			0.99	0.40	0.39		
Disturbed, Intermediate	0.67	0.49	0.54			3.03	**0.03**	**0.04**		
Taxonomic distinctness (Δ*)										
Disturbance	2	2157.80	2.33	0.17	0.18	2	4618.40	30.53	**0.005**	**0.0008**
Location(Dist)	6	978.92	37.94	**0.0001**	**0.0001**	5	153.12	1.14	0.34	0.34
Residual	151	25.80				311	134.03			
Total	159					318				
Pairwise tests	t	P(perm)	P(MC)			t	P(perm)	P(MC)		
Control, Disturbed	2.04	0.08	0.10			11.04	**0.0001**	**0.0001**		
Control, Intermediate	2.10	0.20	0.13			3.43	**0.0001**	**0.04**		
Disturbed, Intermediate	0.05	1	0.97			2.59	0.12	0.07		
**Avg. Taxonomic distinctness (Δ^+^)**										
Disturbance	2	960.02	5.91	**0.05**	**0.04**	2	424.71	8.19	**0.05**	**0.02**
Location(Dist)	6	171.25	9.07	**0.0001**	**0.0001**	5	50.99	0.85	0.52	0.51
Residual	151	18.87				311	59.81			
Total	159					318				
Pairwise tests	t	P(perm)	P(MC)			t	P(perm)	P(MC)		
Control, Disturbed	4.16	**0.04**	**0.01**			3.59	**0.0001**	**0.02**		
Control, Intermediate	2.48	0.10	0.09			3.73	0.10	0.03		
Disturbed, Intermediate	0.07	0.90	0.94			0.03	1	0.98		

Results from PERMANOVA analyses of taxonomic diversity indices. Df = degrees of freedom, MS = Mean square estimate, P(perm) = probability generated from permutation test; P(MC) probability generated from Monte Carlo sampling.

**Figure 8:**
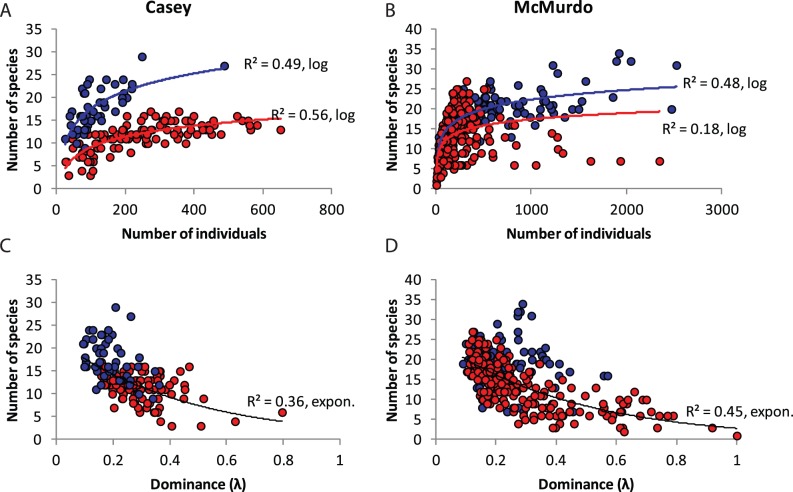
Species abundance and dominance relationships. A & B) The relationship between the number of species and number of individuals in each region; C & D) The relationship between the number of species and dominance in each region. Blue symbols represent control samples, red symbols represent disturbed samples. Type of function used to fit trendline indicated following R^2^ value.

Contrasting patterns of the distribution of individuals among species between Casey and McMurdo and control and disturbed sites can be seen in a Whittaker plot of rank abundance (Fig. 4B). There is a clear difference in evenness of control assemblages with a steeper line for McMurdo (higher dominance) than Casey (more even), and a clear difference between control and disturbed assemblages at each station. The Casey and McMurdo disturbed assemblages have the steepest slopes (Fig. 4B), which are characteristic of an environmental impact [76]. A comparison of dominance using Simpson’s dominance index (λ) indicated significant variability in dominance among locations (Table 3) in both regions, but that in general dominance was greater at disturbed locations (Fig. 8C, D). Pooled reference locations at Casey had a slightly lower dominance (λ = 0.10) than McMurdo (λ = 0.13), but pooled disturbed locations had similar values in each region (Table 3). Dominance also decreased with increasing spatial scale, and was generally largest at the point scale (mean per core at each location), less at the sample scale for each location and lowest at the large area scale for pooled control locations and disturbed locations (Table 3). We observed a significant positive non-linear relationship between the number of individuals and the number of species, that appeared to be different for control and disturbed locations, particularly at Casey (Fig. 8A, B), far more so than at McMurdo. There was also a significant negative correlation between the number of species and dominance per sample, with control samples having generally lower dominance, particularly at Casey.

### Comparison of Community Patterns

**Figure 9:**
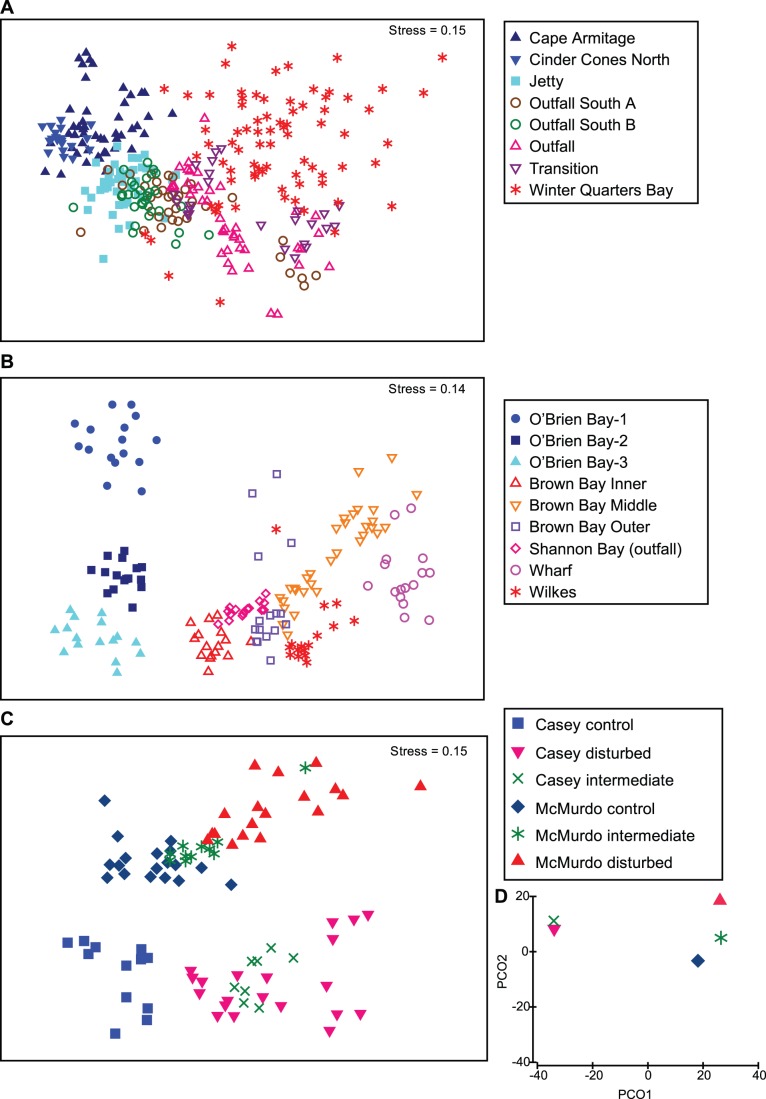
Macrofaunal community patterns in each region. A & B) MDS ordinations of macrofaunal communities at Casey region species level (A) and McMurdo region species level (B); C) Combined family level MDS ordination of macrofaunal communities at Casey and McMurdo, showing control, intermediate and disturbed groups within each region; D) PCO plot of distances among group centroids in C to show relative effect size. Based on square root transformed abundances and Bray-Curtis similarities. PCO1 = 50.8% of total variation, PCO2 = 27.3% of total variation.

Both stations show significant differences in infaunal communities at the species level along a contamination/disturbance gradient, with strong differences between control and disturbed locations (Fig. 9A, B). Differences between control and disturbed locations were more distinct at Casey, with a high level of within-location similarity (clustering of samples in locations in MDS ordination, Fig. 9B) and distinct differences among locations (clearly separated location clusters in MDS ordination), particularly among control locations. At McMurdo there was less separation of different locations in the species level MDS, with more of a gradual change in communities along the contamination gradient (Fig. 9A). This may reflect the higher levels of contamination at McMurdo, as there is a greater spectrum of contamination from control to disturbed with a set of locations that are intermediate in their levels of contamination. This is seen in the species level MDS with the intermediate disturbance locations clustering very closely to the controls (Fig. 9A, B). Additionally it may be influenced by the greater sampling effort at McMurdo and the influence of temporal variation on communities over 10 years.

Casey and McMurdo community patterns were compared directly using family level taxonomic resolution (Fig. 9C). Infaunal communities were different between the two regions with very clear separation between control groups in the MDS ordination (Fig. 9C), indicating that at the family level Casey and McMurdo have different assemblages. The effect of disturbance is also apparent with intermediate and disturbed communities significantly different from controls, but with a response that is in the same plane in the MDS ordination (Fig. 9C). Thus although Casey and McMurdo had differing starting points (as represented in control communities), there were some similarities in the nature of the response in each region at the community level. Intermediately contaminated sites tended to sit in between control and disturbed sites, indicating there is a gradient of response to contaminants, although the varied response of the intermediate category is indicative of its nominal classification. Some locations classified as intermediate more closely resemble disturbed, and some, controls. Importantly the community response to the contamination gradient seen at the species level (Fig. 9A, B) is also apparent at the family level (Fig. 9C).

Two metrics were used to measure the size of the response to disturbance at each station, ANOSIM and distances between group centroids (calculated from the resemblance matrix). Control communities in both regions were compared to intermediate and disturbed groups separately and both regions showed a similar pattern, but the magnitude of the response to contamination/disturbance at Casey was slightly greater than at McMurdo (Table 5). A PCO ordination of distances among group centroids demonstrates a larger difference between control and disturbed at Casey than McMurdo (Fig. 9D). The intermediate group was more distinct from controls at Casey than McMurdo. The size of the difference between the disturbed group at Casey and disturbed group at McMurdo is also similar to the size difference between the control groups in each region, providing further evidence that the degree of the impact is similar at each station (Table 5). The control groups in each region, however, are less variable than the disturbed group, with the intermediate groups least variable overall, probably reflecting the fewer locations (samples) in this category (Table 6).

**Table 5:** Comparison of the magnitude of the effect of disturbance at each station.

	ANOSIM tests				Distance between group centroids		
	Casey		McMurdo		Casey	McMurdo	
	R	P	R	P			
Control vs disturbed	0.71	0.001	0.71	0.001	58		52
Control vs intermediate	0.88	0.001	0.32	0.001	63		33
Intermediate vs disturbed	0.0	0.51	0.25	0.005	19		36
		**R**	**P**				
Casey vs McMurdo (control)		0.90	0.0001			56	
Casey vs McMurdo (disturbed)		0.86	0.0001			63	

Comparison of size of differences between control, intermediate and disturbed groups at family level identification of macrofaunal communities.

**Table 6:** Effects of disturbance on community variability in each region.

Mean deviation from group centroid		
Group	Mean	SE
Casey intermediate	22.6	2.9
McMurdo intermediate	23.1	4.0
McMurdo control	31.0	1.5
Casey control	32.9	2.4
Casey disturbed	34.7	2.0
McMurdo disturbed	36.0	2.1

Comparison of community variability as measured by multivariate dispersion (PERMDISP test).

**Figure 10:**
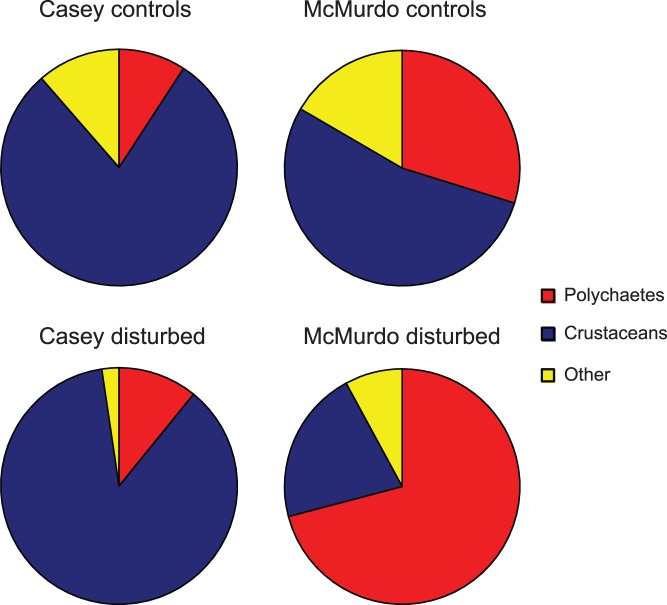
Community composition and effect of disturbance. Relative proportions of polychaetes and crustaceans in infaunal assemblages at Casey and McMurdo, split into control and disturbed locations.

At the phylum level the differences between Casey and McMurdo are due to the relative proportions of polychaetes and crustaceans (Table 7, Fig. 10). Crustaceans dominate the control communities in both regions but are far more dominant at Casey, whereas polychaetes are a relatively minor component of the Casey community but are prominent at McMurdo (Fig. 10). The effect of disturbance, however, is very different in each region. At Casey crustaceans become more dominant, whereas at McMurdo they become less abundant and polychaetes dominate disturbed communities. At McMurdo the proportion of polychaetes is much greater at disturbed locations than controls, whereas at Casey it is very similar at disturbed and control locations (Table 7). Of the crustaceans at Casey, it is the proportion of amphipods which has the greatest change, increasing from an average of 20.7% (SE 6%) of the community at controls to 61.2% (SE 6.9%) at disturbed locations (Fig. 10). The proportion of isopods also increased slightly at disturbed locations in both regions. Ostracods and cumaceans, however, decrease in proportion at disturbed locations in both regions. The proportion of tanaids is approximately the same at both control and disturbed locations at Casey and decreases from control to disturbed at McMurdo. At McMurdo the proportion of all crustacean groups is lower at disturbed locations than controls, except for isopods, which is similar in both (Table 7). Echinoderms decrease slightly at disturbed locations although proportions are generally very low. There is no clear trend for bivalves or gastropods as proportions are very small (Table 7).

**Table 7:** Community composition (by % of major taxonomic groups) in each region and effect of disturbance.

	Casey		McMurdo	
	Control (n = 3)	Disturbed (n = 7)	Control (n = 3)	Disturbed (n = 5)
Polychaetes	12.5±9.5	11.4±9.1	32.1±3.4	67.6±6.1
Oligochaetes	4.0±1.2	0.1±0.03	1.0±0.5	0.7±0.2
Crustaceans	74.4±10.6	86.3±9.0	51±4.6	22.9±5.9
Chelicerates	0.2±0.1	0	0.6±0.3	0.9±0.4
Amphipods	20.9±6.1	62.4±7.2	6.4±1.7	4.4±1.1
Tanaids	13.8±5.4	13.9±4.1	22.5±4.5	6.2±3
Ostracods	33.9±17.7	5.7±3.6	6.8±3.3	0.2±0
Cumaceans	5.0±2.9	0	4.8±1.5	0.3±0.1
Isopods	0.9±0.2	4.2±1.3	10.6±0.5	11.8±1.7
Echinoderms	1.8±1.4	0.1±0.1	0.1±0	0
Bivalves	1.4±1.3	0	0.3±0.1	0.6±0.2
Gastropods	1.9±1.4	0.9±0.6	0.6±0.1	0.5±0.1
Other	3.9±2.3	1.0±0.6	14.3±2.3	6.7±2.1

Mean (and SE) percentage composition of major taxonomic groups. Samples pooled in each location (n = number of locations averaged).

**Figure 11:**
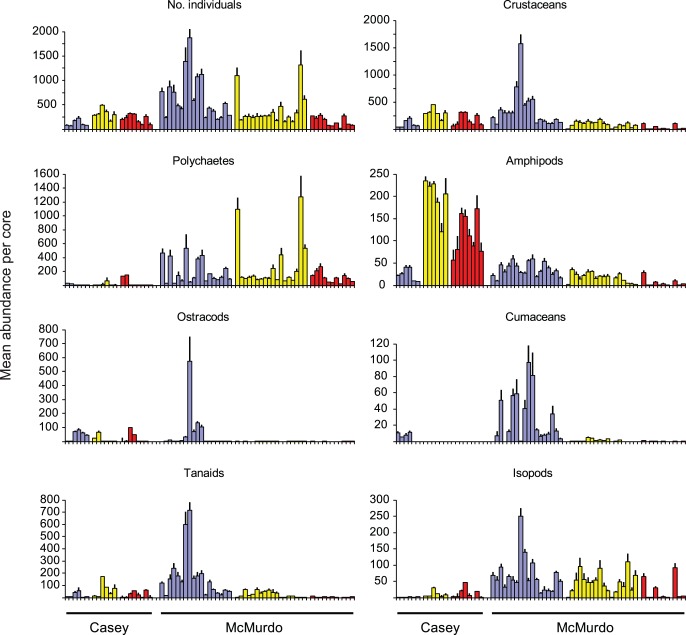
Comparative abundance of key taxa. Comparison of mean abundance per core for major taxonomic groups at Casey and McMurdo. Note Casey data based on 1

Relative abundance is more difficult to compare between the two regions due to the differing sieve mesh sizes used, which underestimates abundance at Casey in comparison to McMurdo. However some relative patterns of abundance are very clear. At McMurdo total abundances were greatest at control sites but at disturbed sites at Casey (Fig. 11). Even if applying a correction factor of 1.5 to Casey estimates (based on the Casey subset sorted at both mesh sizes, see Methods), abundances at the Casey controls are much lower than McMurdo. At disturbed sites however, abundances are similar (Fig. 11), and after applying a 1.5×mesh correction, abundances at disturbed sites at Casey would be greater than McMurdo (excepting several large peaks of abundance at some intermediate disturbance sites at McMurdo). Total abundance of crustaceans shows a similar pattern of differences, with generally greater abundances at McMurdo controls than Casey (after correction for mesh size) and greater abundances at Casey disturbed than McMurdo (even without the correction) (Fig. 11). Amphipods show an incredible difference in abundance at disturbed sites between the two regions, with huge abundances recorded at Casey, which would be even larger with a 0.5 mm mesh. Abundances of amphipods are similar at controls in both regions. Thus, at Casey amphipods increase from control to disturbed while at McMurdo they decrease (Fig. 11). In contrast polychaetes are far more abundant at McMurdo, and although they comprise a greater proportion of disturbed communities they are less abundant than at control and intermediate sites (Fig. 11). Polychaete abundances at Casey, however, are greatest at the disturbed and intermediate sites. Ostracods, cumaceans, tanaids and isopods are far more abundant at McMurdo than Casey, even allowing for a mesh size correction. Ostracods and cumaceans both show the same pattern of greater abundance at control sites in both regions (Fig. 11).

There were several taxa which could be considered potentially useful indicators of disturbance in Antarctic soft-sediment assemblages (Table 8). At the species and family level there were some similarities in the taxa comprising control and disturbed assemblages at Casey and McMurdo, for example cumaceans and ostracods both were more abundant at controls than disturbed sites in both regions (Table 8). At disturbed sites there were also similar taxa present, in particular three polychaete families (Table 8). Thus even though there were big differences in overall assemblage composition, there were similar characteristic taxa in control and disturbed assemblages in both regions. There were also some contrasting patterns for some taxa, for example amphipods of the genus *Orchomenella* were very abundant at disturbed sites at Casey but extremely rare at McMurdo disturbed sites and in low abundances at controls.

**Table 8:** Taxa with potential for use as indicators of disturbance.

Opportunist taxa	Sensitive taxa
**Polychaetes**	**Polychaetes**
Orbiniid polychaetes (C,M)	Spionid polychaetes (M)
*Haploscoloplos kerguelensis* (M)	*Spiophanes tcherniai (M)*
*Leitoscoloplos mawsoni* (C)	Paraonid polychaetes (C)
Capitellid polychaetes (C,M)	Nephtyid polychaetes (C)
*Capitella capitata* (M)	**Crustaceans**
*Capitella perarmata* (C)	Leuconid cumaceans (C,M)
Dorvelleid polychaetes (C,M)	*Eudorella splendida* (M)
*Ophryotrocha puerilis* (M)	*Eudorella gracilior* (C)
*Dorvelleid* sp. II (C)	*Leucon antarcticus* (C)
Cirratulid polychaetes (M)	Amphipods
*Tharyx* sp. (M)	*Monoculodes curtipediculus(C)*
Hesionid polychaetes (M)	Philomedid ostracods (C,M)
*Gyptis* sp. (M)	*Philomedes spp.* (M)
**Crustaceans**	*Philomedes charcoti* (C)
Lysianassid amphipods (C)	*Scleroconcha gallordoi* (C)
*Orchomenella franklini* (C)	**Actiniaria**
*Orchomenella pinguides* (C)	*Edwardsia meridionalis* (M)
Exoedicerotid amphipods	**Molluscs**
*Methalimedon nordenskjoeldi* (C)	Rissoid gastropods (C,M)
	Gastropod sp. A: *Onoba/Eatoniella* (M)
	*Skenella paludinoides*(C)
	*Eatoniella cf. kerguelensis* (C)
**Contrasting patterns**	
*Austrosignum grande* (Isopoda): High abundance McMurdo control and intermediate, high abundance Casey disturbed	
*Orchomenella franklini/pinguides:* High abundance McMurdo control, high abundance Casey disturbed	
Cirratulid polychaetes: High abundance McMurdo disturbed, not present Casey disturbed	

Taxa indicative of disturbed and control conditions. M = McMurdo, C = Casey, Indicator taxa = abundant at disturbed, Sensitive taxa = abundant at controls or not present at disturbed.

## Discussion

Antarctic research stations have the potential to severely impact marine benthic communities. The accumulation of contaminants in marine sediments causes changes in community composition and loss of biodiversity. Responses of benthic communities to anthropogenic disturbance show some regional differences, largely due to differing regional community composition. There are, however, some common responses and species which are potential indicators of, or sensitive to, disturbance which may aid in the rapid detection of impacts in other areas. Assessing the relative impact of stations depends on a clear understanding of sampling effort and potential methodological biases. Such impact assessments will lead to improved environmental management and inform decisions regarding potential remediation actions, while contributing to a better understanding of regional biodiversity for conservation and management purposes.

At both Casey and McMurdo there is clear evidence of human impacts in macrobenthic communities, with contamination of marine sediments matched with differences in community composition, reduced biodiversity and characteristic indicator taxa. A common feature in response to disturbance in both regions was reduced diversity at disturbed sites, whether measured as observed species richness, estimated total species richness or taxonomic diversity/distinctness. Contamination clearly has an impact on biodiversity in Antarctic soft sediment communities as has previously been demonstrated at Casey [28]. The nature of the community response, however, is quite different in each region. The response of macrofaunal communities at McMurdo was similar to that seen in other parts of the world [77], with an increase in the proportion of polychaetes to become the dominant component at McMurdo (disturbed sites range 46–82%, mean 68±6%), whereas they decreased at Casey (disturbed sites range 0.2–65%, mean 11±9%). Globally, polychaetes are known to characterise contaminated sediments, whether influenced by organic enrichment or other contaminants such as metals [77]–[79]. At McMurdo the disturbed sites are organically enriched but they are also contaminated with anthropogenic pollutants such as metals and hydrocarbons [19], [25], [26]. Some polychaete species are tolerant to pollutants and can produce detoxifying secondary metabolites and proteins in response to contaminants [80]–[83]. These responses are usually confined to several families of polychaetes with opportunistic life histories, and at McMurdo the prominent families at disturbed sites included dorvelleids, capitellids, orbiniids, cirratulids and hesionids, which were also seen to respond positively in experimental manipulations of organic matter [80], [84]. While an increase in the proportion of polychaetes was not seen at Casey, there were several polychaete families characteristic of disturbed sites in both regions (Table 8), including Capitellidae, Dorvelleidae and Orbiniidae. These families have been found to dominate or have increased abundances in disturbed areas in other regions [77], [85]–[89] including the Arctic [90]. Abundances of these three families increased at some disturbed sites at Casey but overall polychaetes at Casey did not respond in the manner described in other regions and studies. This may be due to their low abundances and regionally low representation in the community, with potentially only a small pool of source populations to recruit from. Local recruitment is likely to be the dominant source and have a strong influence on assemblage composition (e.g. [91]). Isolation by regional oceanographic currents, such as has been shown at McMurdo [92], may also contribute to dominance of local recruitment.

A major difference in the response to disturbance was in the proportion of crustaceans, which decreased at disturbed sites at McMurdo but increased at Casey, mainly due to amphipods. Experiments by Lenihan et al. [80] at McMurdo showed that in general crustaceans were negatively affected by increasing metal (Cu) concentrations and this has also been observed in other experimental work [93]–[95]. Lenihan et al. [80] also demonstrated a decrease in crustaceans in response to increasing organic enrichment. Oakden et al. [95] described behavioural avoidance by phoxacephalid amphipods to sediment organically enriched by sewage effluent. This difference in crustacean response between the two stations may relate to the levels of contamination, with McMurdo having generally higher concentrations of contaminants. It may also reflect the generally greater dominance of crustaceans in Casey communities. Of the amphipods at Casey it was the family Lyssianassidae that had highest abundances at disturbed compared to control sites. Lyssianassid amphipods are known scavengers [96] and respond positively to organic enrichment in the deep sea [97]–[99] and have been found in large abundances in disturbed areas in the Arctic [85]. Stable isotope ratios of nitrogen indicate that lyssianassids found at Casey are at an intermediate trophic level comprising deposit feeders and scavengers [100], [101]. The increase in the abundance and proportion of lyssianassids at Casey may be a response to increased organic carbon content of sediments at disturbed sites. As metal concentrations are generally lower at Casey than McMurdo, lyssianassids may not be challenged by the above-background levels of metals present in the sediments at Casey. Alternatively other contaminants present at McMurdo, such as persistent organic compounds [21], may not be present at Casey, although this remains to be tested.

Some taxa showed a similar sensitivity to contamination in both regions, being either absent from contaminated sites or in very low abundances. Cumaceans and ostracods are two examples, and may prove to be effective indicators of disturbance in Antarctica. Whether it is a response common to all Antarctic cumacean and ostracod species remains to be determined. In other parts of the world cumaceans, for example, have been found to be both sensitive to disturbance such as organic enrichment [102] as well as indicative of these conditions [103], depending on the species. Ostracods, however, are generally sensitive to pollution and disturbance in marine habitats [104] and our results support this. Echinoderms were also significantly reduced in abundance or absent from disturbed sites and previous Antarctic work has found them to be negatively affected by metals but to increase in response to organic enrichment [80]. There were also other similarities in the fauna comprising the control communities in the two regions. Several amphipod families characterised the control assemblages including Oedicerotidae, Lyssianassidae and the phoxacephalid species *Heterophoxus videns*. Other taxa characteristic of control communities in both regions included cumaceans, philomedid ostracods, oligochaetes, Rissoidae gastropods and cirratulid polychaetes (Table 8).

One of the aims of this study was to identify potentially useful indicator species whose general utility could be in tested in future work or used in the development of indices or biocriteria to assess environmental conditions. Biological assessment criteria are increasingly being used to assess ecological impacts in estuarine and coastal ecosystems in other regions of the world, e.g. by the US Environmental Protection Agency [105] and the European Water Framework Directive [106], [107], of which benthic invertebrates are a key component [89]. The development of such methods in Antarctica is in its very early days as knowledge of the fauna and their responses to the environment is very limited. It is of great importance, however, that attempts are made to identify common responses and potential indicators to disturbance. Further field studies from other regions of Antarctica are needed to test such generalizations, in addition to species specific ecotoxicological studies assessing tolerance or sensitivities to contaminants [108]. Very little is known of the pollution tolerance or adaptation to pollution for Antarctic marine invertebrates and field observations are limited.

This study found that small Antarctic stations such as Casey can have impacts of equal or greater severity on marine benthic communities as large, more disturbed and contaminated stations such as McMurdo. McMurdo has a much larger human population and some sites are considered to be as contaminated as heavily industrialized estuaries in other parts of the world [13]. However the magnitude of the response to disturbance in each region is fairly similar, if not larger at Casey compared to McMurdo. Casey has a greater loss of biodiversity at its disturbed sites than McMurdo, although McMurdo has a greater level of dominance than at Casey. McMurdo is atypical and almost unique in comparison to other Antarctic stations. Casey is more representative of the size of many Antarctic stations. On the basis of the findings of this study it is likely many coastal Antarctic research stations have some level of environmental impact on local marine ecosystems. It is too early to propose that the size of the impacts at all coastal stations would be similar, but clearly even comparatively low levels of contamination and disturbance could cause large effects as demonstrated in this study. This study provides evidence that Antarctic marine benthic communities may be particularly sensitive to disturbance.

The Casey and McMurdo regions have considerably different soft-sediment macrobenthic communities, primarily in the relative proportions of polychaetes and crustaceans. McMurdo is more typical of macrobenthic assemblages worldwide, with a larger proportion of polychaetes (control sites at McMurdo 26–38%, mean 32±3%) compared to Casey (controls 2–30%, mean 13±10%). Polychaetes tend to dominate soft sediment assemblages globally, usually comprising at least 30–60% of total abundance [109]–[114]. Casey is unusual in its almost complete numerical dominance by crustaceans (controls 54–89%, mean = 74±11%), whereas they were less dominant at McMurdo (controls 42–57%, mean 51±5%).

The unusual community composition at Casey may be due to large scale regional processes. Antarctic nearshore benthic ecosystems have highly seasonal primary production and are thought to be food limited [84]. Regional hydrographic conditions around McMurdo are fairly well described and it is an area considered to be relatively eutrophic due to the current flowing into the eastern side of McMurdo Sound, bringing with it the spring phytoplankton bloom from further north [92], [115]. These eutrophic conditions may account for the extremely high abundances (up to 250,000 individuals m^−1^) recorded at reference locations in the McMurdo region. In contrast little is known of regional hydrographic conditions around Casey. Casey has greater sediment organic carbon content than McMurdo, which has been confirmed in other studies [116], and indicates a highly productive ecosystem with possibly greater food availability for benthic fauna. Casey is also further north than McMurdo on an area of Antarctic coastline which is more open, and sea ice breaks out in some areas over winter and earlier in summer than McMurdo (although not at the sites used in this study), which may also contribute to higher in-situ primary production. The Windmill Islands/Casey region is situated in Vincennes Bay, which is a katabatic polynya [117] that may enhance primary production in the region. The distinct differences between macrofaunal communities at Casey and McMurdo may be related to these broad scale oceanographic differences. The unusual community composition at Casey (dominance by crustaceans), however, is unlikely to be related to local environmental properties such as sediment type as they are similar to other regions of Antarctica. There is nothing outwardly unusual in local physical environmental conditions to indicate any potential cause for such unusual phyletic composition at Casey. One possible biological and habitat difference between the two regions is the presence of macroalgae. The Casey region has large areas of macroalgal habitat in areas which have less sea ice (these areas were not part of this study), whereas McMurdo has very little or no macroalgal cover, due to longer ice cover over the region and less available light as it is further south. The addition of this major food source in the Casey region [100], [101] may have profound influences on benthic community composition and diversity. The positive influence of primary productivity on Antarctic benthos has been emphasised by several studies [15], [92], with sediment carbon content increasing β-diversity within locations [15]. Food quality may also be important as the proportion of nitrogen in sediments has been shown to be important at regional scales [15].

While the two areas may have some differences in primary production levels and ice cover at a regional scale, the actual sites used in this study at both regions have similar levels of ice cover duration and therefore are likely to have similar levels of physical disturbance by ice. Ice scour has not been quantified at the locations in this study but our observations indicate that they have similar ice duration affording them similar levels of protection from scour, which has been shown to be a major structuring force in other areas of Antarctica such as the Peninsula region [118], [119]. The sites used at Casey represent the far end of an ice cover gradient which extends from sites with no ice for long periods of the year (excluded from this study) to sites covered by ice for most of the year [52], which were used in this study. There is less of a gradient at McMurdo and most sites are similar in their duration of ice cover and are at the ice-covered end of the Casey spectrum.

At the regional level and within regions at scales greater than 1 km, Casey is more diverse than McMurdo, as measured by numerical species richness, however, indices of taxonomic distinctness [68], [120], [121] were greater at McMurdo suggesting greater taxonomic diversity. These measures, which describe the path length or average taxonomic distance between two randomly selected organisms through the phylogeny (Δ and Δ* based on quantitative data, and average taxonomic distinctness Δ^+^, based on presence/absence), have been suggested by Warwick and Clarke [122] as being more easily comparable measures of biodiversity due to their lack of dependence on sampling effort or differences in taxonomic rigor. A greater taxonomic distinctness at McMurdo indicates greater taxonomic diversity or less relatedness among all species overall, or a wider spread of diversity across phylogenies. One possible explanation for this is the broader range of depths at McMurdo, with closer proximity of nearshore, shallow water habitats to deep shelf habitats and ice shelves. McMurdo is also at higher latitude than Casey which may contribute to a greater range of physical environmental conditions and a wider range of adaptations and thus taxonomic diversity. Habitat diversity has been found to have a positive effect on β-diversity in Antarctic infaunal communities [15]. Taxonomic distinctness, however, cannot be seen as a substitute for species richness, but as an additional piece of information on species diversity, and Casey has a greater number of species overall than McMurdo, with less sampling effort. Warwick and Clarke [122] found that Δ^+^ is also useful in identifying degraded habitats and at the point scale at Casey and McMurdo this appears to be the case, however at the scale of large area, there is no difference between control and intermediate or disturbed habitats. This may be due to the differing nature of the disturbances across sites, affecting different fauna at different sites, but when all disturbed sites are combined this effect is nullified.

There is much to be gained in comparisons involving data from different studies, including the ability to make generalizations and increase overall understanding of ecological responses to environmental changes. The opportunity to sample widely separated regions using the same methods, at similar times and for a single unified purpose are exceedingly rare and difficult to organize, involving different countries, funding bodies and logistic operations. An alternative method is to opportunistically use the results from independent studies. Comparisons of environmental impacts using community data from different studies will usually encounter a range of difficulties, for example, due to differing sampling methods, but as demonstrated here, as long as potential sources of bias are identified, valid comparisons are possible.

Sampling effort is a major consideration in inter-study comparisons. Sampling effort, either the total number of samples taken or the total cumulative area they represent, has a strong influence on estimates of species richness. In regional comparisons using different studies, sampling effort and species richness can easily be assessed by comparisons of species-area curves. Species richness should ideally be compared as close to the asymptote of species-area curves as possible [31], however if sampling effort is unequal this may not be possible, but if one or more curves in the comparison is nearing an asymptote a valid comparison can be made. Casey’s greater diversity and the strong effect of disturbance on diversity are best demonstrated in these curves. Diversity is likely to be underestimated at Casey due to the greater sampling effort at McMurdo, comprising a longer period of time and greater number of samples. Importantly the Casey and McMurdo samples cover a similar spatial extent, as this can have a large effect on estimates of species diversity [30]. Realistic community description for comparisons among samples, community patches, and different communities is best done in a known spatial context [35]. At small spatial scales (the point and sample level) McMurdo appears to be more diverse, but this may be due in part to underestimation of point-scale diversity at Casey with a larger mesh size (1 mm vs. 0.5 mm). Above this scale however, we have demonstrated that mesh size effects are unlikely to be significant. Previous work at Casey has demonstrated that community patterns were the same when using either 1 mm or 0.5 mm data [123]. Thus the fauna passing through a 1 mm sieve are only smaller individuals, not different species, but due to the larger number of individuals more species are encountered at a point (core) or sample (group of cores at a site) scale. Casey’s greater diversity thus is even more significant when considering the lesser sampling effort than at McMurdo.

Another potential confounding factor in comparisons of impacts is species level identifications of highly diverse macrofauna, particularly when working in regions where there is a paucity of taxonomic information or resources such as Antarctica. One approach to dealing with this challenge is the use of coarser taxonomic resolution, such as family as opposed to species. Using coarser levels there is less chance of incorrectly identifying particular taxa, whereas at the species level there is often great uncertainty, particularly when considering the recent spate of cryptic species being described in this area [124]–[126]. In general there is little loss of community information when moving from species to family level when examining patterns related to environmental gradients or habitat heterogeneity [51], [127]. We demonstrate that the same is true for the Antarctic, whereby patterns of community composition in response to anthropogenic contamination gradients were preserved when going from species to family level.

Direct comparison of contaminant levels should be done with caution due to the different analytical methods used. For example sediment metal data based on a total extraction may actually obscure some low level anthropogenic contamination and this is highly likely to be the case at McMurdo. Research done at Casey comparing these two methods indicated a varying degree of difference depending on the metal examined, for example copper concentrations in total digests were approximately twice those observed in partial digests [61]. However copper levels in total digests at Casey contaminated sites are still well below levels seen at McMurdo at contaminated sites. Standardized approaches to measuring contaminants in sediments are recommended, although unlikely to occur in the short term due to the historical nature of measurements within regions. This highlights the importance of studies which compare the effects of different methodologies on measuring contaminants, enabling inter-study comparisons to be made of disturbance levels. The relative effects of contaminants between regions are not solely related to contaminant concentrations, however, but also depend on other physical and biological factors.
